# Epidemiological analysis of the early 38 fatalities in Hubei, China, of the coronavirus disease 2019

**DOI:** 10.7189/jogh-10-011004

**Published:** 2020-06

**Authors:** Yifei Chen, Meizhen Zhao, Yifan Wu, Shuang Zang

**Affiliations:** 1 School of Nursing, China Medical University, Shenyang, Liaoning, China; 2 Nursing Department, Tongji Hospital Affiliated to Tongji Medical College, Huazhong University of Science and Technology, Wuhan, Hubei, China

## Abstract

**Background:**

Since the emergence of coronavirus disease 2019 (COVID-19) in Hubei province of China by the end of 2019, it has burned its way across the globe, resulting in a still fast-growing death toll that far exceeded those from severe acute respiratory syndrome (SARS) in less than two months. As there is a paucity of evidence on which population is more likely to progress into severe conditions among cases, we looked into the first cluster of death cases, aiming to add to current evidence and reduce panic among the population.

**Methods:**

We prospectively collected the demographic and clinical data of the first 38 fatalities whose information was made public by the Health Commission of Hubei province and the official Weibo account of China Central Television news center, starting from 9 January through 24 January 2020. The death cases were described from four aspects (gender and age characteristics, underlying diseases, the time course of death, symptoms at the incipience of illness and hospital admission).

**Results:**

Among the 38 fatalities, 71.05% were male, and 28.95% were female, with the median age of 70 years (interquartile range (IQR) = 65-81). Persons aged 66-75 made up the largest share. Twenty-five cases had a history of chronic diseases. The median time between the first symptoms and death was 12.50 days (IQR = 10.00-16.25), while the median time between the admission and death was 8.50 (IQR = 5.00-12.00) days. In persons aged over 56 years, the time between the first symptoms and death decreased with age, and so did the time between the admission and death, though the latter increased again in persons aged over 85 years. The major first symptoms included fever (52.63%), cough (31.58%), dyspnea (23.68%), myalgia and fatigue (15.79%).

**Conclusions:**

Among the death cases, persons with underlying diseases and aged over 65 made up the majority. The time between the first symptoms and death decreased with the advanced age. In all the age groups, males dominated the fatalities.

Since a cluster of unexplained cases of pneumonia, which was later identified as a novel coronavirus infection, surfaced in December 2019 [1], the virus, initially named as 2019 novel coronavirus (2019-nCoV) by the World Health Organization (WHO), and later as Severe Acute Respiratory Syndrome Coronavirus 2 (SARS-CoV-2) by the International Committee on Taxonomy of Viruses, has ravaged in Wuhan, and swiftly spiraled to other areas in China. At a breakneck pace, the virus has also left over 190 countries under siege, prompting WHO to declare COVID-19 a pandemic on 11 March 2020. As the number of infected cases keeps rocketing up, the death toll of the coronavirus disease 2019 (COVID-19) outbreak has overtaken that of the severe acute respiratory syndrome (SARS) during the 2002-2003 epidemic. The COVID-19 outbreak has wreaked havoc on all sectors in China, resulting in city lockdown, traffic restrictions, work shutdown, and school cancellation, etc., first in Wuhan, then later in many other cities. Many countries have imposed travel restriction, suspended flights, and barred entry of Chinese nationals. The sudden shock of the COVID-19 has had a significant impact on the Chinese economy [2]. Containment of virus transmission has become a top priority to global public health security.

Researchers have been racing against time since the outbreak of the COVID-19, as little was known regarding COVID-19 virus initially. Two viral genome studies had indicated that the novel virus is closely related to SARS-CoV (one research revealing 79.5% and the other 89.1% nucleotide similarity, respectively) [3,4], which is reminiscent of the calamitous SARS outbreak 17 years back. Aside from viral genome studies, researchers also looked into the clinical features and epidemiologic characteristics of COVID-19 cases. Clinical manifestation of COVID-19 ranges from mild symptoms (low-grade fever, fatigue, sore throat, etc.) that resemble a common cold [5], to severe and even fatal respiratory diseases such as acute respiratory distress syndrome [6]. A study that collected more than 70 000 cases across China also reported asymptomatic cases of COVID-19 virus infection, accounting for 1.2% of total confirmed cases [7]. After emergence, the virus spread rapidly through human-to-human transmission [8], which was substantiated by a modeling study from Los Alamos National Laboratory indicating the median basic reproductive number (R0) for COVID-19 virus was 5.7 (95% confidence interval (CI) = 3.8-8.9) [9]. The mechanism behind the high infectivity of the COVID-19 virus could be explained by a study revealing that COVID-19 virus spike glycoprotein had around 10 to 20-fold higher affinity with angiotensin converting enzyme II (ACE2) receptor that is widely distributed in human organs, than SARS-CoV spike glycoprotein [10]. In addition, the COVID-19 virus spreads mainly from person-to-person contacts via respiratory droplets or contact with infected surfaces or objects [11]. With a combination of high infectivity and easy transmission, the COVID-19 virus poses a great threat to anyone who has close contacts with an infected person, especially within families and to frontline medical staffers [12]. To make things worse, transmission from asymptomatic patients was confirmed by a case report covering a German businessman being infected by his asymptomatic Chinese business partner from Shanghai [13]. WHO issued a warning against possible transmission of COVID-19 virus from infected people before they developed symptoms. These findings have raised concerns across the globe, sounded the alarm of a dire situation, and prompted authorities to ramp up quarantine measures.

To what extent COVID-19 kills remains vague, as literature in terms of case fatality rate is in scarcity. Based on the data compiled by WHO, the overall case fatality rate of COVID-19 globally was initially estimated at around 2% [14], similar with an overall case fatality rate of 2.3% from a study which collected more than 70 000 cases in mainland China as of 11 February 2020 [7]. However, the two case fatality rate figures were much lower than the result yielded from the study of Wang et al. on a case series of 138 consecutive hospitalized COVID-19 patients (mortality: 4.3%) in a hospital in Wuhan, China [15]. The reason that the study of Wang et al. had higher case fatality rate can be attributed to the large scale of the infection in the epicenter of the outbreak (more than 40 000 cases in early February 2020), the heavily strained medical system, the lack of protective suits and medical equipment (such as masks, goggles, gloves, and disinfectants). Based on the fact that the death toll of COVID-19 topped that of the SARS outbreak during 2002-2003 in less than two months, COVID-19 virus infection will deal a more substantial blow to the globe and can be more fear-mongering, than SARS. A look into deaths cases may provide more information to the public and sooth panic, as studies suggested that misinformation and inadequate information contribute to unnecessary public panic and subsequent undesirable responses [16,17]. As there is a paucity of evidence on which population is more likely to progress into severe conditions among COVID-19 cases, here, we poured over the first batch of 38 death cases whose information were made public by Health Commission of Hubei province as of 24 January 2020, one day into city lockdown in Wuhan, with the purpose to add a new facet to current evidence.

## METHODS

### Source of data

Data of COVID-19 death cases in Hubei were extracted prospectively from the website of Health Commission of Hubei province [18] and the official Weibo (China’s equivalent of Twitter) account of China Central Television news center [19], starting from 9 January 2020, when the first deceased patient was reported, through 24 January 2020, when the 38^th^ was registered. Since 25 January 2020 the number of death cases has been surging, and the Health Commission of Hubei province has stopped making public the information of death cases. Therefore, data collection was terminated at that point.

### Statistical analysis

Microsoft Excel 2016 (Microsoft, Redmond, WA, USA) and SPSS 23.0 software (IBM Corp., Armonk, IL, USA) was used for data analysis. The death cases were described from four aspects (gender and age characteristics, underlying diseases, death time distribution, and symptoms at the incipience of illness and hospital admission). Frequencies (%) and median (interquartile ranges [IQR]) were used to describe the data.

## RESULTS

### Gender and age characteristics

**Figure 1:**
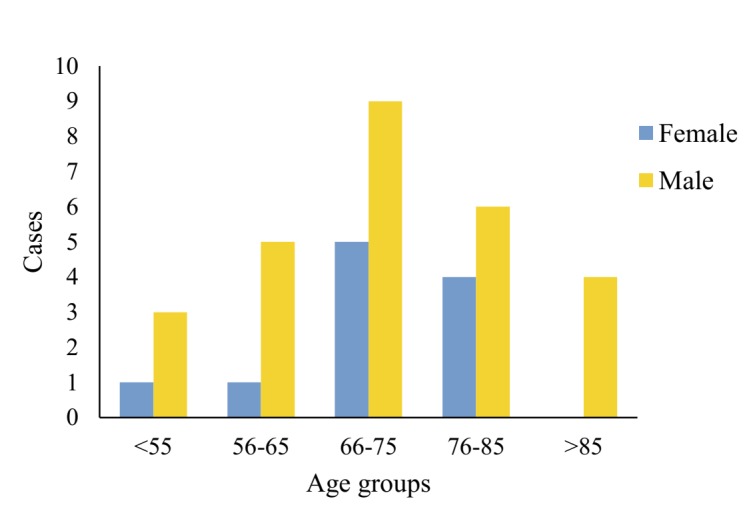
Distribution of 38 fatalities by genders and age groups.

As of 24 January 2020, the overall case fatality rate for COVID-19 was 5.3% in Hubei. Among the fatalities, there were 27 males, and 11 females, with a male to female ratio of 2.45:1. The youngest age was 36 years, and the oldest age was 89 years, with the median age being 70 years (IQR = 65-81). The median age for females and males both stood at 70, though IQR ranged from 66 to 80 for the former, and from 65 to 81 for the latter, respectively. Distribution of 38 fatalities by genders and age groups was shown in **Figure 1**. There were 14 cases aged 66-75 years, making up the largest share of 36.84%. Coming next was 10 cases aged 76-85, accounting for 26.31%. The same pattern was found for genders, with 66-75 years forming the largest share of 33.33% in males, vs 45.46% in females alone, and 76-85 years the second largest (22.22% in males, vs 36.36% in females alone).

### Underlying diseases

Among the death cases, 25 had underlying diseases, including 16 males and nine females, accounting for 65.78% of the total. There were 15 cases of hypertension, 11 cases of diabetes, four cases of coronary heart disease, three cases of chronic bronchitis, two cases of cerebral infarction, and two cases of Parkinson disease. Other diseases included chronic obstructive pulmonary disease, tuberculosis, frequent ventricular premature beats, colon cancer, gallstone, cirrhosis, chronic renal insufficiency, fracture, hip replacement, etc., all keeping a tally of one case, respectively (**Table 1**). Among all the death cases, 17 had one or two underlying diseases, and eight had more than three underlying diseases.

**Table 1:** Statistics of the underlying diseases of 38 dead cases*

Underlying diseases	Cases
Hypertension	15
Diabetes	11
Coronary heart disease	4
Chronic bronchitis	3
Cerebral infarction	3
Parkinson disease	2
Cerebromalacia	1
Frequent ventricular premature beat	1
Coronary stenting	1
Chronic renal insufficiency	1
Chronic obstructive pulmonary disease	1
Tuberculosis	1
Schizophrenia	1
Colon cancer	1
Gallstone	1
Cirrhosis	1
Fracture	1
Hip replacement	1
Primary myelofibrosis	1

*Note: Some death cases have two or more underlying diseases before death.

### Distribution of death time

Among the 38 fatalities, the first case died on 9 January, and the last in the batch died on 24 January 2020, with the period stretching 15 days, during which, the death toll didn’t show apparent regularity.

To understand the evolution of death, we defined the first symptom day fell on the date on which the patients started to feel their symptoms. We described the period between the first symptom day and the death date as days from the first symptom to death and the period between the date of admission and death as days from admission to death.

The median time from the first symptom to death was 12.50 days (IQR = 10.00-16.25). As for male dead patients, the median time from the first symptom to death was 13.00 days (IQR = 11.00-17.00), and for the females, the median time from the first symptom to death was 11.00 days (IQR = 9.00-14.00).

The median time from admission to death was 8.50 days (IQR = 5.00-12.00). As for male dead patients, the median time from admission to death was 9.00 days (IQR = 5.00-13.00), and for the females, the median time from admission to death was 7.00 days (IQR = 5.00-11.00).

**Figure 2:**
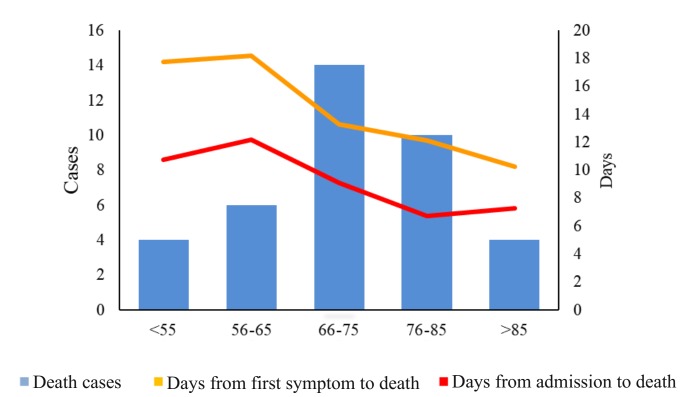
Days from the first symptom to death and days from admission to death among different age groups.

Days from the first symptom to death tailed off over the age groups of 56-65, 66-75, 75-85, and >85, while the days from admission to death had a similar pattern over the age groups of 56-65, 66-75, 75-85, but rebounded in persons aged over 85 (**Figure 2**). 66% of the cases died within nine to 15 days since they felt the first symptoms.

### The main symptoms

Fever and cough were the main reported symptoms at the onset of illness among the 38 early death cases. Twenty patients first complained of a fever, 12 of coughs, 9 of dyspnea, 6 of chest tightness, 6 of myalgia and fatigue, accounting for 52.63%, 31.58%, 23.68%, 15.79%, and 15.79%, respectively. Other symptoms included headache, dizziness, chills, and intermittent diarrhea, each keeping a tally of one case.

Fever and dyspnea were the main reported symptoms at hospital admission among the 38 early death cases. Twenty-five patients complained of a fever, 23 of dyspnea, 16 of coughs, 10 of chest tightness, and 6 had the complaints of myalgia and fatigue, accounting for 65.79%, 60.53%, 42.11%, 26.32%, and 15.79%, respectively. Other symptoms are shown in **Table 2**.

**Table 2:** The symptoms of 38 deaths.

Symptoms	Symptoms at the onset of illness (n = 25)	Symptoms at hospital admission (n = 38)
Fever	20	25
Cough	12	16
Dyspnea	9	23
Chest tightness	6	10
Myalgia and fatigue	6	6
Delirium	0	2
Headache	1	1
Dizziness	1	1
Chills	1	1
Intermittent diarrhea	1	1

## DISCUSSION

As of 24 January 2020, the initial overall case fatality rate in Hubei Province reached 5.3%. Later on, newly reported cases in China saw a sharp rise, but the overall case fatality rate has dwindled. As of 11 February, the overall fatality rate in Hubei province was 2.9% [7], which was far lower than the results of our study. The later declining overall case fatality rate was on one part attributed to the effective treatment of COVID-19 as thousands of medical workers from other parts of China poured into Hubei Province to aid their fellow workers battling the coronavirus. On the other part, there was a substantial shortage of test kits at the early stage of the COVID-19 outbreak, making it challenging to identify the infected cases [20]. Afterward, the test kits were supplied in a large amount, making the number of confirmed patients grow significantly. Besides, with a continuous flow of medical resources and personnel into the epicenter and the sweeping screening of infected persons in the communities, the infections were identified and admitted to the hospitals (including Fangcang shelter hospitals) speedily, reducing the possibility of becoming severe and preventing the widespread of the coronavirus in communities. We discussed the epidemiological characteristics of 38 cases in the early stage of the disease from the following four parts.

### Gender and age characteristics

71.05% of the deaths were male, considerably more than female, which is consistent with the findings of Wang W et al. [21]. Single-cell sequencing of COVID-19 virus receptors at Tongji University found that Asian men were more likely to be infected with COVID-19 virus [22], and a study of 8866 cases nationwide also found that the death rate of men was more than three times that of women [23]. The reason that male dominated the fatalities could be explained by the fact that percentage of ACE2 level in men is higher than in women [23], rendering men more susceptible to COVID-19 virus.

In addition, COVID-19 virus-infected people tend to be older ones [24]. In a recent *Lancet* article (15 February 2020) [6], 53% of the confirmed cases had chronic underlying diseases, and the median age was 55.5 years, indicating that the middle and old aged patients with chronic underlying diseases were more likely to contract the COVID-19 virus.

### Underlying diseases

From experience, we can see that patients with chronic underlying diseases are indeed more likely to have disease deterioration or even death. Among the death cases, persons with underlying diseases and aged over 65 made up of the majority. Hence, we developed a speculation that COVID-19 could worsen in elderly persons with underlying diseases and even more easily progress to death. This is mainly due to the dwindling immunity in the elderly, especially in those with underlying diseases, which directly renders senior people more likely to be in a state of frailty and more vulnerable to infections [25], and subsequently leads to worsening of the disease [26].

Among the death cases, persons with hypertension and /or diabetes made up the largest share, which could be explained by the fact that hypertension and diabetes top the chronic disease chart in China [27]. ACE2 is a crucial regulator of the renin-angiotensin system, and plays a regulatory role in the central regulation of blood pressure and cardiovascular function and could become an attractive target for the treatment of hypertension [28,29]. COVID-19 virus uses the receptor ACE2 to enter into target cells [30], precisely the same as SARS-CoV. Turner et al. [31] found that SARS-CoV infection affects the function of ACE2, so we speculate that the COVID-19 virus will also impair the function of ACE2, and then manipulate the regulation of blood pressure, and have a negative impact on patients with hypertension. On the other hand, hypertension can cause vascular damage. In patients with hypertension, increased vascular stiffness and decreased elasticity are common, followed by vascular remodeling and stenosis [32]. The pathological results of patients with COVID-19 showed that pulmonary vessels endothelial swelling, luminal stenosis and occlusion, leading to acute lung dysfunction [31]. The coexistence of hypertension and COVID-19 is a very unfavorable factor to induce lung dysfunction, which is prone to aggravate the condition and even result in death. ACE2 gene can also be expressed in the pancreatic islets. A study showed that the binding of SARS-CoV to ACE2 damages islets and causes acute diabetes [33]. COVID-19 virus may also exert such a negative effect on islets through the same mechanism. In persons with preexisting diabetes, the damage of islets by COVID-19 virus could be more severe, and even fatal [4,34,35]. In addition, ACE2 is rich in the lungs, heart, kidney, intestine, and testicles, etc. Once COVID-19 virus gains entry into the human body, more organs could be attacked by the virus through blood circulation over time [36]. Therefore, early diagnosis of COVID-19 before it progresses into severe conditions is an important measure for older people who have developed a fever and respiratory symptoms [24]. Other measures including reducing chances of exposure to infected cases (eg, banning visits to nursing home residents, avoiding gatherings), early isolation and treatment of symptomatic confirmed cases can be beneficial to the elderly population, especially those with preexisting underlying diseases.

### Time distribution of death

In this study, the number of deaths did not show obvious regularity with time within two weeks. In addition, the COVID-19 virus infection rate has been spiking up since 20 January. According to the data of the National Health and Health Commission [37] and the results from our study, we make the following speculation: the cases gradually became infected around the end of December according to a median 7-day incubation period and a median 12.5-day period from the first symptom to death [20]. If we take the later reported maximum incubation period of 38 days into account [38], a considerable part of them may have been infected in November. Besides, the difference in immune resistance between different genders and ages is also an important reason for the irregular distribution of the time of death [39].

However, from **Figure 2****,** it can be concluded that with the increase of age, the days from hospital admission to death and the days from the first symptom to death gradually reduced indeed, which shows that COVID-19 poses a great threat to elderly patients [40]. Our study also shows that the days from hospital admission to death rebounded in persons aged over 85 years. Since there were only 4 cases aged over 85 years, the finding needs further validation from long-term, large-scale cohort studies.

### The main symptoms

Studies have indicated that viral infection in the early stage mainly shows upper respiratory tract infection, manifested as fever, headache, and cough [41]. Huang et al. found a similar result that 98% of the patients with COVID-19 experienced fever, 76% had a cough, and 55% had dyspnea as the first symptom, respectively [42]. However, among the deaths up to 24 January, 52.63% had a fever, 31.58% had a cough, and 23.68% had dyspnea, as the first symptom. It can be seen that not all infected cases have high body temperature as the first symptom, and the temperature change of old people is not very significant compared with young ones even when they have infectious diseases [43]. Among the severely infected elderly, 20% ~ 30% of them have no fever or slow response to fever, which is often a sign of poor prognosis [44], and hinders early detection of infection and brings more potential risks to the elderly. Therefore, the early repeated examination is a valid response [45]. However, symptoms changed at the time of admission, with 65.79% of patients showing fever, 60.53% dyspnea, 42.11% cough, indicating dyspnea became the second major symptom. As for the COVID-19 virus infection, severe patients will have chest discomfort, progressive dyspnea, or acute respiratory distress syndrome symptoms [46], which indicates the aggravation of the disease. As a result, the proportion of dyspnea symptoms was slightly higher than other symptoms in our deaths. Also, although there were cases with limb myalgia and fatigue, headache, and other initial symptoms came in a small quantity, it does not mean that COVID-19 cases presenting the symptoms are in mild condition. There is still the possibility of progression to death, which should arouse the vigilance of medical staff [47].

The emergence of a new infectious disease poses a particular challenge to epidemiologic research, as identifying the characteristics of the disease and infection prevention and control of an epidemic is a step-by-step process. During the period from 17 to 20 January 2020, the number of confirmed cases of COVID-19 increased 10-fold [48], indicating high infectivity of the novel coronavirus [49]. Such a disease needs to be contained, or at least the spread of it needs to be reined in time. Otherwise, the medical system will face enormous pressure, and a large number of infected patients will inevitably die due to the lack of timely treatment. During the COVID-19 outbreak, it is necessary to strengthen the training of medical personnel from all levels of medical institutions, especially those serving at hospitals designated as the treatment center for the disease. At the same time, it is necessary to invest a multitude of resources in outpatient and emergency departments to detect patients to improve the treatment conditions and the capacity to house severe cases. Since the elderly and people with underlying diseases are most vulnerable to the attack of coronavirus, and often have serious consequences [50], it is urgent to ramp up protection and prevention measures for the elderly, especially those with chronic underlying diseases. It also warns us that in the face of an unknown disease, the protection of vulnerable people is essential.

This study has some limitations. First, the data of this study came from the panel data of the official website of the Health Commission of Hubei province, so the clinical information of the cases collected is limited. Second, as our study focused on the deaths in the early stage of the outbreak, and the fatalities constitute only a tiny fraction of the overall still-hiking death toll, the specific relationship between male and female, and the variations in the time window from onset to death, and from admission to death among different age groups needs more large-scale studies .

## CONCLUSIONS

This study represents characteristics of the earliest deaths in the early outbreak for COVID-19 using the panel data in China. We found that the elderly and people with underlying diseases dominated the death cases, and the days from the first symptom to death gradually shortened with the increase of age. In different age groups, the male fatality count is higher than that of female.
